# Transcriptomic and Metabolomic Analyses Provide Insights into the Growth and Development Advantages of Triploid *Apostichopus japonicus*

**DOI:** 10.1007/s10126-022-10093-4

**Published:** 2022-02-05

**Authors:** Jiahui Xie, Yi Sun, Yue Cao, Lingshu Han, Yuanxin Li, Beichen Ding, Chuang Gao, Pengfei Hao, Xin Jin, Yaqing Chang, Jian Song, Donghong Yin, Jun Ding

**Affiliations:** 1 Key Laboratory of Mariculture & Stock Enhancement in, Ministry of Agriculture and Rural Affairs, North China’s Sea, Dalian Ocean University, Dalian, Liaoning, People’s Republic of China 116023; 2 Ningbo University, Ningbo, Zhejiang People’s Republic of China 315211

**Keywords:** Triploid *Apostichopus japonicus*, Transcriptome, Metabolome, Growth, Aestivation

## Abstract

Polyploid breeding is widely used in aquaculture as an important area of new research. We have previously grown *Apostichopus japonicus* triploids with a growth advantage. The body length, body weight, and aestivation time of triploid and diploid *A. japonicus* were measured in this study, and the transcriptome and metabolome were used to examine the growth advantage of triploids *A. japonicus*. The results showed that the proportion of triploid *A. japonicus* with a body length of 6–12 cm and 12–18 cm was significantly higher than that of diploid *A. japonicus*, and triploid *A. japonicus* had a shorter aestivation time (39 days) than diploid (63 days). We discovered 3296 differentially expressed genes (DEGs); 13 DEGs (for example, *cyclin-dependent kinase 2*) related to growth advantage, immune regulation, and energy storage were screened as potential candidates. According to Gene Ontology (GO) enrichment analysis, DEGs were significantly enriched in the cytoplasm (cellular component), ATP binding process (molecular function), oxidation–reduction process (biological process), and other pathways. According to the Kyoto Encyclopedia of Genes and Genomes (KEGG) enrichment data, DEGs were significantly enriched in ribosome production and other areas. We discovered 414 significant differential metabolites (SDMs), with 11 important SDMs (for example, nocodazole) linked to a growth advantage. SDMs are significantly enriched in metabolic pathways, as well as other pathways, according to the KEGG enrichment results. According to a combined transcriptome and metabolome analysis, 6 DEGs have regulatory relationships with 11 SDMs, which act on 11 metabolic pathways together. Our results further enrich the biological data of triploid *A. japonicus* and provide useful resources for genetic improvement of this species.

**Supplementary Information:**

The online version contains supplementary material available at 10.1007/s10126-022-10093-4.

## Introduction

Polyploidy, which was first proposed by Winkler (1916), refers to the occurrence of three or more genomes in each somatic cell (Joana et al. 2018). Most polyploids naturally exist in fish but, at present, artificial polyploidy is frequently used. Polyploid breeding first appeared in the artificial induction of triploid carp by Makino and Ojima (Ren et al. 2018). Since then, polyploid breeding of aquatic animals has been widely used in fish and shellfish. Polyploid breeding has good market value in shellfish. Guo invented the production method for tetraploid mollusks (oysters, scallops, clams, mussels, and abalones) in 1995, and proposed the method of producing triploid by mating tetraploid and diploid (Guo and Allen 1994; Guo et al. 1996). Compared with diploid oysters, triploid oysters have the advantages of large size, fast growth (Guo et al. 2009; Guo 2021), and high nutritional value (Qin et al. 2018). At present, polyploid breeding has been applied or studied in more than 40 types of fish and more than 20 types of economic shellfish and crustaceans (Song et al. 2004).

*Apostichopus japonicus* (Echinodermata: Holothuroidea: Aspidochirotida: Stichopodidae) is an important economic echinoderm, and is a species known to be of the best quality among the more than 20 types of edible sea cucumbers. However there have been few studies on the polyploid breeding of echinoderms, except that Chang successfully induced triploid and tetraploid *A. japonicus* using cytochalasin B and 6-dimethylaminopurine (6-DMAP), and examined the inducing drug concentration, treatment time, treatment start time, and the survival rate of larvae (Chang and Xiang 2002). Ding then proposed the method of inducing triploid *A. japonicus* by hydrostatic pressure (Ding et al. 2007). Han et al (2021) used transcriptome and methylation to study the role of methylation changes of different genes in triploid *A. japonicus*. However, the molecular mechanism of controlling the dominant traits such as fast growth and short aestivation time of triploid *A. japonicus* is not clear. Therefore, we expect to determine the significantly different genes, metabolites, and metabolic pathways related to triploid *A. japonicus* which participate in growth immunity, and understand the relationship between different genes and different metabolites.

In recent years, exploring the growth and development (Sun et al. 2017; Xing et al. 2021), immune function (Li et al. 2018; Shi et al. 2020), and phylogeny (Zhao et al. 2020; Carmona et al. 2017) of aquaculture animals using various omic techniques have gradually become a research hotspot (Chen et al. 2020). The combined analysis of transcriptome and metabolome is widely used to reveal the molecular mechanism in organisms (Wang et al. 2020; Liu et al. 2019; Kong et al. 2020). Therefore, we used the combined transcriptome and metabolome sequencing technology to analyze triploid *A. japonicus*. The different gene expression patterns of triploid and diploid *A. japonicus* have been identified. Some differential genes and metabolites in triploid *A. japonicus* that stimulate growth, metabolism, immune regulation, and protein synthesis have been found and verified, and differential genes and differential metabolites were jointly analyzed. The results in the present study will enrich the basic biological data on triploid *A. japonicus*, and provide resources for the future breeding of triploid *A. japonicus*.

## Materials and Methods

### Culture and Detection of *A. japonicus*

The triploid *A. japonicus* induced by hydrostatic pressure came from the same batch as the diploid control group produced at the Ministry of Agriculture and Rural Affairs’ North Key Laboratory of Marine Aquaculture at Dalian Ocean University, and all were 1.5-year-old *A. japonicus*. During the experiment, the breeding conditions were as follows: water temperature 14 ± 1.5 °C, salinity 30 ± 1, and pH 7.0. During the breeding process, the water was changed every 2 days, and feeding was done once a day (feed formula: sea mud, compound feed, spirulina powder, purslane powder). We used flow cytometry (Sysmex, Japan) to determine the ploidy of *A. japonicus*, and the procedure was the same as Han’s (Han et al. 2021).

### Analysis of Growth Characters

**Fig. 1:**
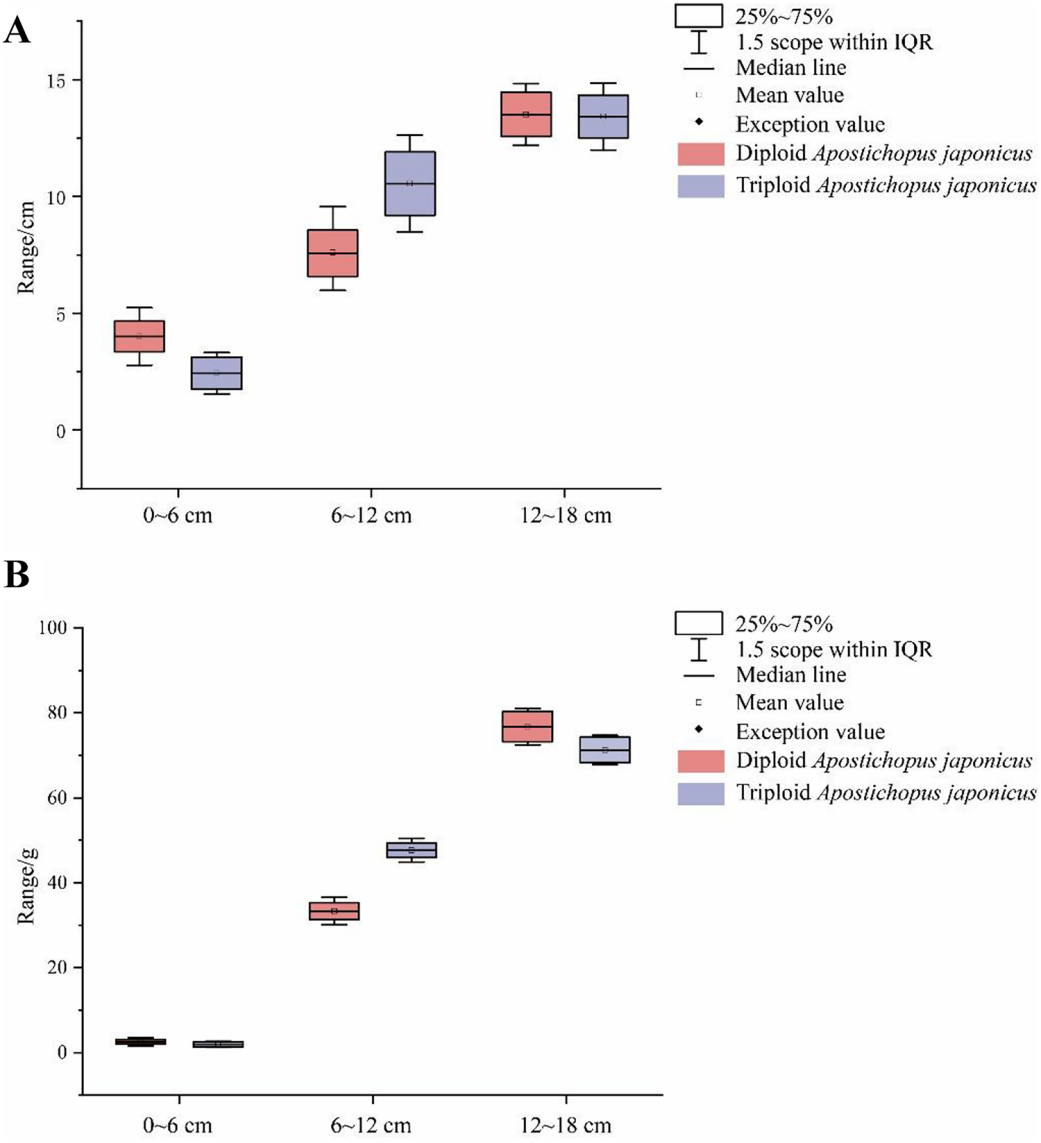
Box diagram of body length (**A**) and body weight (**B**) of triploid and diploid *Apostichopus japonicus*

To determine the ploidy of *A. japonicus*, a tiny amount of tube feet was cut and dissolved in 1.2-ml cell lysate (cystatin UV precise P/05–5002, Japan) in a 1.5-ml centrifuge tube. Flow cytometry was used to detect *A. japonicus* after the cell components had been destroyed (Han et al. 2021). Flow cytometry detected 2682 *A. japonicus*, including 510 triploid *A. japonicus* and 2172 diploid *A. japonicus*. The body lengths of all *A. japonicus* were then measured. To make comparisons easier, we divided the body length of *A. japonicus* into three groups (0–6 cm, 6–12 cm, and 12–18 cm) and compared its proportion in each group (Fig. 1) (Table S1). We compared the aestivation times of triploid and diploid *A. japonicus* which is defined as the time starting with the cessation of feeding, body contraction, and inactivity and ending with relaxation, activity, and feeding (Table 1).

**Table 1:** Comparison of aestivation time between triploid *Apostichopus japonicus* and diploid *Apostichopus japonicus*

Group	Observation time	Aestivation date	End of aestivation	Aestivation duration
Triploid group	2019	July 27	September 3	38 days
Diploid group	2019	July 12	September 5	55 days
Triploid group	2020	July 24	September 1	39 days
Diploid group	2020	July 9	September 10	63 days

### Sample Preparation

In 2020, three *A. japonicus* were dissected in each experimental group, yielding a total of 22 groups of samples, 6 of which were subjected to transcriptome sequencing (3 triploid *A. japonicus*, 3 diploid *A. japonicus*) and 16 of which were subjected to metabolomic analysis (8 triploid *A. japonicus*, 8 diploid *A. japonicus*). The body wall tissues were quickly frozen in liquid nitrogen and kept at −80 °C in a refrigerator.

### RNA Extraction and Quality Control

Total RNA was extracted from the body wall tissue of the *A. japonicus* using a Mirvana™ miRNA Isolation Kit, Ambion (USA), and the purity and concentration of total RNA were detected using the Agilent 2100 and Nanodrop (Severino et al. 2013). RNA detected with a purity between 2.0 and 2.1 was stored at − 80 ℃. Gene expression was measured by the FPKM value (Li et al. 2021; Han et al. 2021).

### RNA-Seq and Screening for Key Differential Genes and Data Analysis

The Cutadapt program (https://cutadapt.readthedocs.io/en/stable/,version:cutadapt-1.9) was used to eliminate adaptor contamination from reads (command line: cutadapt -a ADAPT1 -A ADAPT2 -o out1.fastq -p out2.fastq in1.fastq in2.fastq -O 5 -m 100). After removing the low-quality bases and uncertain bases, we utilized the HISAT2 software (https://daehwankimlab.github.io/hisat2/,version:hisat2-2.0.4) to map reads to the genome. StringTie (http://ccb.jhu.edu/software/stringtie/,version:stringtie-1.3.4d.Linux × 86 64) with default parameters was used to build the mapped readings of each sample. Then, using the gffcompare program (http://ccb.jhu.edu/software/stringtie/gffcompare.shtml,version:gffcompare-0.9.8.Linux × 86 64), all transcriptomes from all samples were combined to rebuild a full transcriptome. StringTie and ballgown (http://www.bioconductor.org/packages/release/bioc/html/ballgown.html) were used to estimate the expression levels of all transcripts and perform expression level for mRNAs by calculating FPKM once the final transcriptome was created. The differentially expressed mRNAs were selected with Fold Change > 2 or Fold Change <  − 2 and *p*-value < 0.05 by R package edgeR (Robinson et al. 2010) (https://bioconductor.org/packages/release/bioc/html/edgeR.html) or DESeq2 (Pertea et al. 2015) (http://www.bioconductor.org/packages/release/bioc/html/DESeq2.html). We improved our understanding of the biological functions of the genes by using the Gene Ontology (GO) and Kyoto Encyclopedia of Genes and Genomes (KEGG) databases; enrichment analysis of GO and KEGG was performed using GSEA (gene set enrichment analysis) (https://www.omicstudio.cn/login). All RNA clean data were submitted to the Short Read Archive (SRA) Sequence Database at the National Center for Biotechnology Information (NCBI) (Accession No. PRJNA760261).

### qRT-PCR Validation and Analysis of DEGs

Thirteen DEGs were validated by qRT-PCR to further validate the RNA-Seq results (Ge et al. 2020). Primer Premier 6.0 was used to create primer syntheses for the differential genes (Table 2). The 2^−ΔΔCt^ method was used to calculate the relative expression of DEGs (Arocho et al. 2006).

**Table 2:** Primer sequences of the tested genes used in the quantitative RT-PCR analysis

Gene name	Primer (5′ → 3′)
CYTB	F TGACAGGACCGCTACGAAAGAGG R AAAGTTTTCTTGGGGCCGGAAGG
CDK2	F CACTGCTGAAGGAGTTGGACCATG R ATCGGCTGGAGACCTTGACTGG
CDC45	F AACAGACGAAGATCACGCAACCTC R CAAGTTCAGGAAGTGGCGGGATTC
ORC1	F CAGTGACGATGAGGAGGAGGAGAG R GGAGTTGCTGCTTTAGCGGAGAC
GAMT	F GGAAGGGGAAGACTGTAAGAGC R AATAATACGCACAAGAGGCAGG
UGT	F GGCAGTGTTGGATCCGTTGATGG R CGGTGGTGAAGTCGGCATTGG
PGM	F GCAGCAGCCAATCAGGTGAGG R GTGAGTATAATGCCGCCGGTAGC
MRPs	F TCTTAGACAACGGGTGGCAAT R AAGAATGTCGGGTGGTCCTG
GP96	F CAAGTCGAGGAGGATGGTGAAAGC R CCAGCAAAGGCAGCGGAGTC
HSC70	F GCCTACCAGAGAATTGCCACATCC R ACATCGGGCACTCTTTGTTCTACC
HK	F CGGGGAAGTAATTTCAGAGTCC R ACGGCAGCGATCAATGCT
CBRs	F TCACAGGTTCCAACAAGGGC R CTGAGTACATCTCCCCTCTGCC
Histidine methyltransferase	F TCAATACGAAAGCCACCAAATG R CTTTACCGTCCTCTTTCGTCG
DPD	F TCCAGATGCCTCAAGTGCG R CAATCCACTGTCCGTCATCGT

### Analysis of the Metabolicome, Liquid Phase Analysis, and Mass Spectrometry

For each sample, 100 mg of *A. japonicus* body wall was weighed, ground with liquid nitrogen, and treated before loading the metabolome according to Yu et al. (2018). Before loading, all metabolic samples were kept at − 80 °C. Ultra-high pressure liquid phase and a TripleTOF5600plus (SCIEX, UK) high-resolution mass spectrometer are used for analysis during liquid phase analysis and mass spectrometry. Its model and parameter settings are similar to those of Wang et al. (2019). Quality control (QC) samples were added before, during, and after the test. Prepare QC samples by combining the same number of samples to determine the instrument status and evaluate the stability of liquid chromatography-mass spectrometry (LC–MS/MS) (Li et al. 2019).

### Screening of Key Metabolites

The *q*-value by BH correction was calculated using univariate analysis (Fold Change) and the *t* test. PLS-DA VIP values were combined with multivariate statistical analysis to identify metabolites that were differentially expressed. Differential ions fulfilled the following criteria: ratio ≥ 2 or ratio ≤ 1/2, *q*-value ≤ 0.05, and VIP = 1 or higher. The KEGG enrichment pathway was used to investigate the importance of enrichment (Zheng et al. 2019).

### Combined RNA-Seq and Metabolome Analysis

The process of discovering transcriptome and metabolome associations can be broken down into three steps: (1) the KEGG metabolic pathway connects the transcriptome and metabolome, (2) data screening for statistically significant differences in data and regulatory relationships, and (3) GO and KEGG enrichment analysis.

## Results and Analysis

### Growth Characteristics of Triploid and Diploid *A. japonicus*

The flow cytometer results showed that the peak value of diploid *A. japonicus* was around 200 and that of triploid *A. japonicus* was around 300 (Fig. S6). This outcome is consistent with that of Han (Han et al. 2021). The measurement results revealed that the proportions of triploid *A. japonicus* in the 12–18- and 6–12-cm groups were higher than those in the diploid control group (Fig. 1 and Table S1). Aestivation of triploid *A. japonicus* cultured in 2016 was observed. The results showed that aestivation duration of triploid *A. japonicus* in 2019 was 38 days, while that of diploid *A. japonicus* in the control group was 55 days. The triploid *A. japonicus* aestivation duration in 2020 was 39 days, while that of diploid *A. japonicus* in the control group was 63 days (Table 1). According to these data, triploid *A. japonicus* had a shorter aestivation time and greater growth advantage.

### Overview of Transcriptome Sequencing

**Fig. 2:**
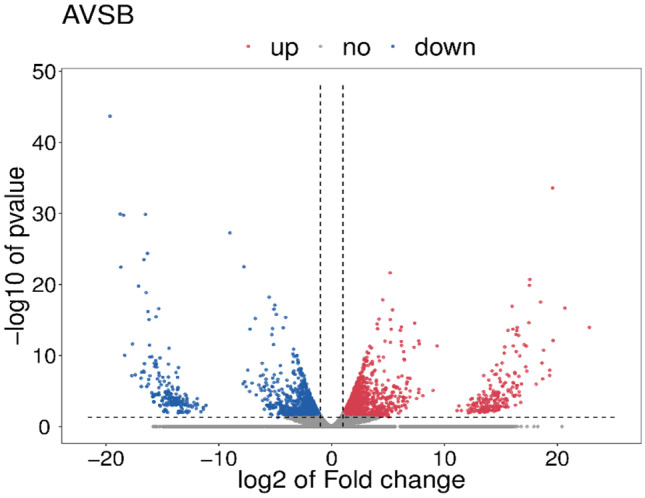
Volcano plot of differentially abundant genes. Note that the red dots represent up-regulation and the blue dots represent down-regulation. **A** Triploid *Apostichopus japonicus* and **B** diploid *Apostichopus japonicus*

The transcriptome data obtained were analyzed. All samples were sequenced independently. In diploid *A. japonicus*, 117.5-M raw reads were obtained, while in triploid *A. japonicus*, 141.83-M raw reads were obtained. The diploid obtained 106.79-M clean reads after removing the redundant data, while the triploid obtained 125.24-M clean reads. Diploid *A. japonicus* transcriptome Q30 was 98.01%, and triploid *A. japonicus* transcriptome Q30 was 98.16%, demonstrating the reliability of transcriptome data. When the GC content of diploid and triploid was compared, it was discovered that diploid had a GC content of 41–43% and triploid had a GC content of 42% (Table S2). A total of 3296 differential genes were identified, with 1856 being up-regulated and 1440 being down-regulated (Fig. 2).

### DEG Enrichment Results in GO and KEGG

The top 50 GO terms with the highest enrichment of differential genes were enriched and analyzed (Fig. S1). The results revealed that the most significantly enriched GO term in the cellular component was cytoplasmic, with 348 DEGs (up: 244, down: 104). The nucleus was the second most important location, with 314 DEGs (up: 233, down: 81). Among the molecular functions, the ATP binding process had the most differential genes (172 DEGs) (up: 126, down: 46). The second process was metal ion binding, which had 144 DEGs (up: 79, down: 65). The oxidation–reduction process was the most important item in the biological process classification, with 96 DEGs (up: 55, down: 41). Proteolysis was the second most important process, accounting for 90 DEGs in total (up: 36, down: 54).

Pathway items with more than 21 differential genes were screened, and the top 20 were chosen based on the − log10 *p*-value corresponding to each item in descending order. Through differential gene screening and enrichment analysis, 1114 differential genes were discovered to be enriched in 224 KEGG signaling pathways, 32 of which were significant (*p* < 0.05). DEGs were significantly enriched in pathways related to ribosome biogenesis in eukaryotes (62 DEGs; up: 57, down: 5), protein processing in the endoplasmic reticulum (52 DEGs; up: 45, down: 7), ECM-receptor interaction (50 DEGs; up: 21, down: 29), RNA transport (44 DEGs; up: 41, down: 3), and lysosomes (43 DEGs) (Fig. S2).

### Screening for Key Differential Genes

**Fig. 3:**
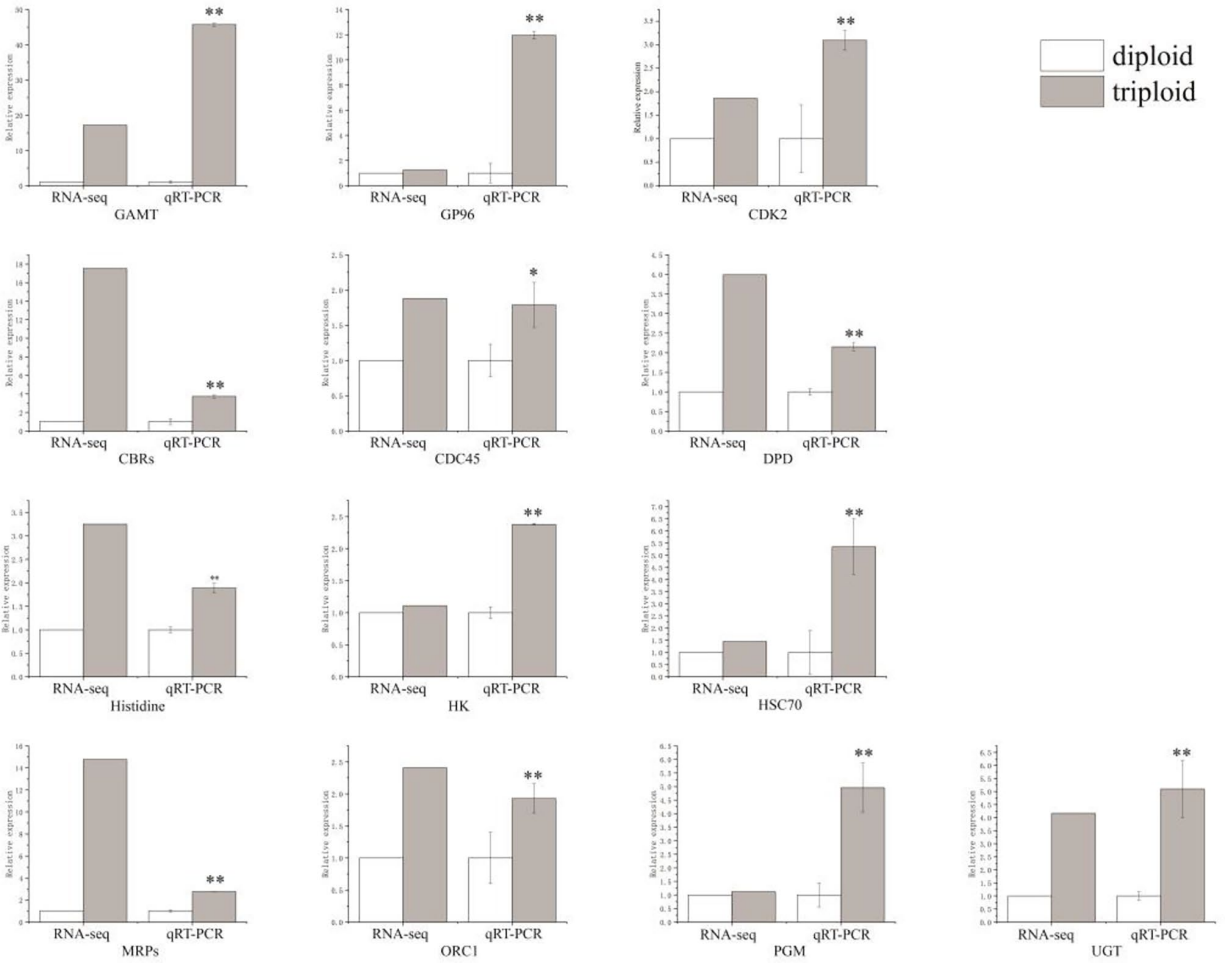
Quantitative RT-PCR validation of differentially expressed genes. *Significant differences at *p* < 0.05 vs. diploid (triploid). **Highly significant differences at *p* < 0.01 vs. diploid (triploid). The results are expressed as mean ± SEM and one-way ANOVA

According to the gene functions annotated by GO and KEGG, we discovered 13 key significantly different genes that may participate in the mechanism of growth advantage in triploid *A. japonicus*, and 13 genes were up-regulated, namely, *cyclin-dependent kinase 2* (*CDK2*), *cell division cycle 45* (*CDC45*), *origin recognition complex subunit 1* (*ORC1*), *multi-resistance associated proteins* (*MRPs*), *hexokinase* (*HK*), *UDP glucuronosyltransferase* (*UGT*), *phosphoglucomutase* (*PGM*), *carbonyl reductase* (*CBRs*), *guanidinoacetate methyltransferase* (*GAMT*), *glycoprotein 96* (*GP96*), *heat-shock cognate protein 70* (*HSC70*), *histidine methyltransferase*, and *dihydropyrimidine dehydrogenase* (*DPD*) (Figs. 3 and 7; Table 2).

### qRT-PCR Validation

The 13 key DEGs identified by transcriptome analysis were validated using qRT-PCR (Table 2). The verification results agreed with the RNA-Seq results (Fig. 3), demonstrating the reliability of the experimental results.

### Partial Least Squares Discriminant Analysis (PLS-DA) of Comparative Groups

**Fig. 4:**
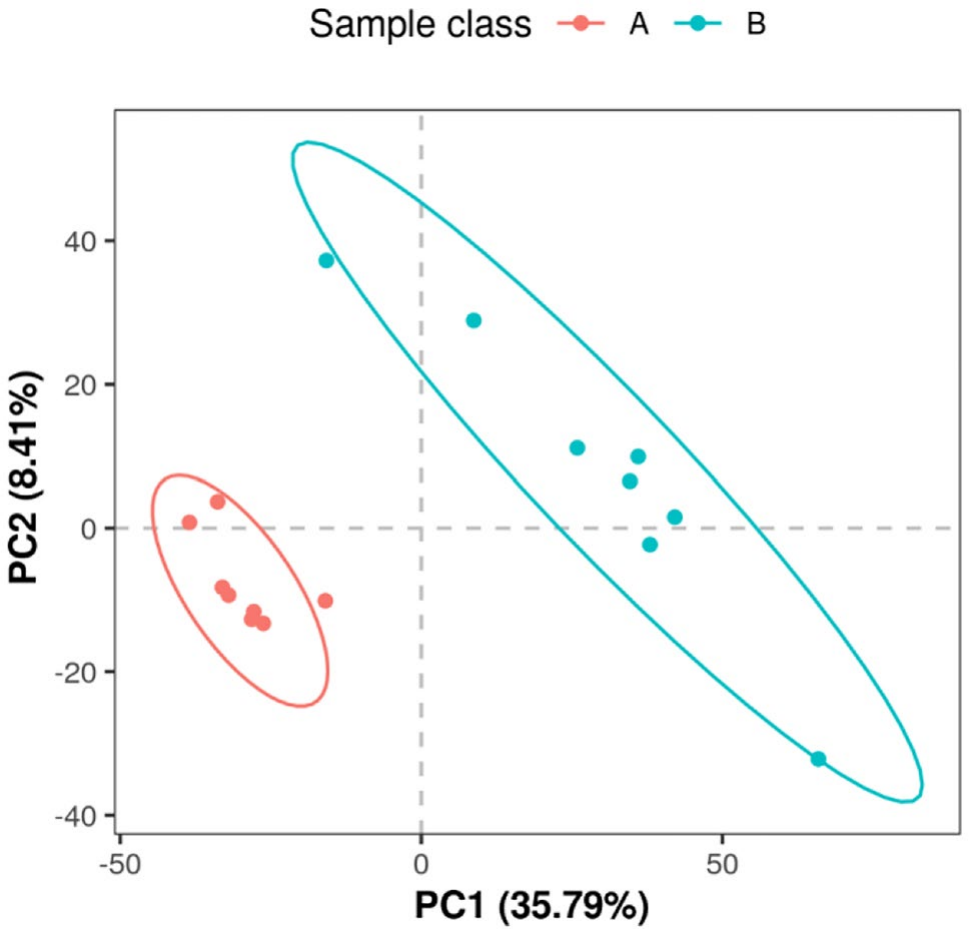
Comparison group PLS-DA analysis. Note that each point in the figure represents a sample, and the similarities and differences among all samples are reflected in the tendency of separation and aggregation of samples in the figure. The aggregation of points indicates that the observed variables have a high degree of similarity, while the dispersion of points indicates that the observed variables have obvious differences. **A** Triploid *Apostichopus japonicus* and **B** diploid *Apostichopus japonicus*

A PLS-DA model was established between each pair of groups using VIP ≥ 1.0 as the screening condition in order to perform principal component analysis on the identified metabolic ions (Fig. 4). In this study, samples from the triploid and control groups were found to have a high degree of dispersion, with no overlap between the two groups of samples. The results were reliable, and the samples were analyzed further by metabolome.

**Fig. 5:**
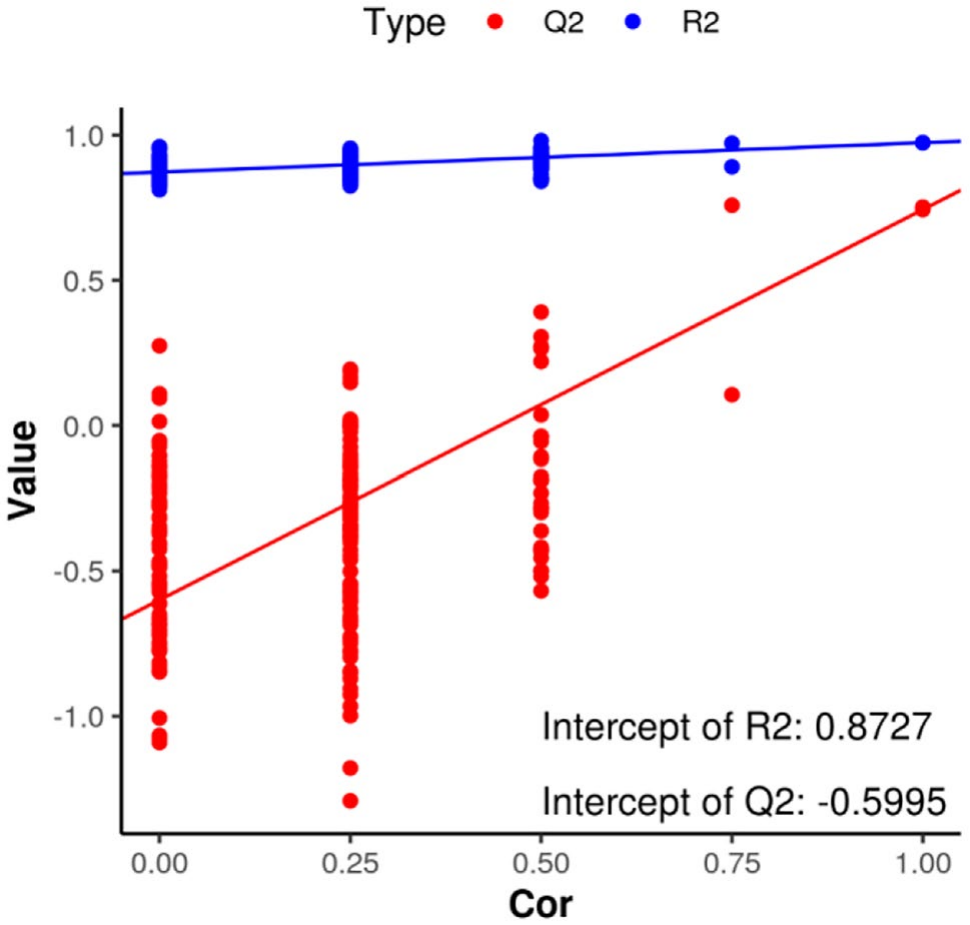
Triploid and diploid *Apostichopus japonicus* replacement test. Note that *Q*_2_ represents the prediction rate of the model and *R*_2_ represents the interpretation rate of the model. *Q*_2_ =  −0.56510 < 0, *R*_2_ = 0.8762

During model validation, linear regression was performed between the original classification *Y* matrix and the *Y* matrix of *N* different permutations with *R*_2_*Y* and *Q*_2_*Y*, and the obtained regression line and *Y*-axis intercept values were *R*_2_ and *Q*_2_, respectively. They were used to determine whether the model was over-fitting. *Q*_2_ =  −0.56510 < 0, *R*_2_ = 0.8762. This demonstrated that the model did not involve over-fitting and that the differential metabolite analysis was sufficiently accurate (Fig. 5).

### Differential Metabolite Statistical Analysis and Screening

A total of 414 metabolites were identified, including 306 positive metabolites (up: 44, down: 262) and 108 negative metabolites (up: 6, down: 102).

**Fig. 6:**
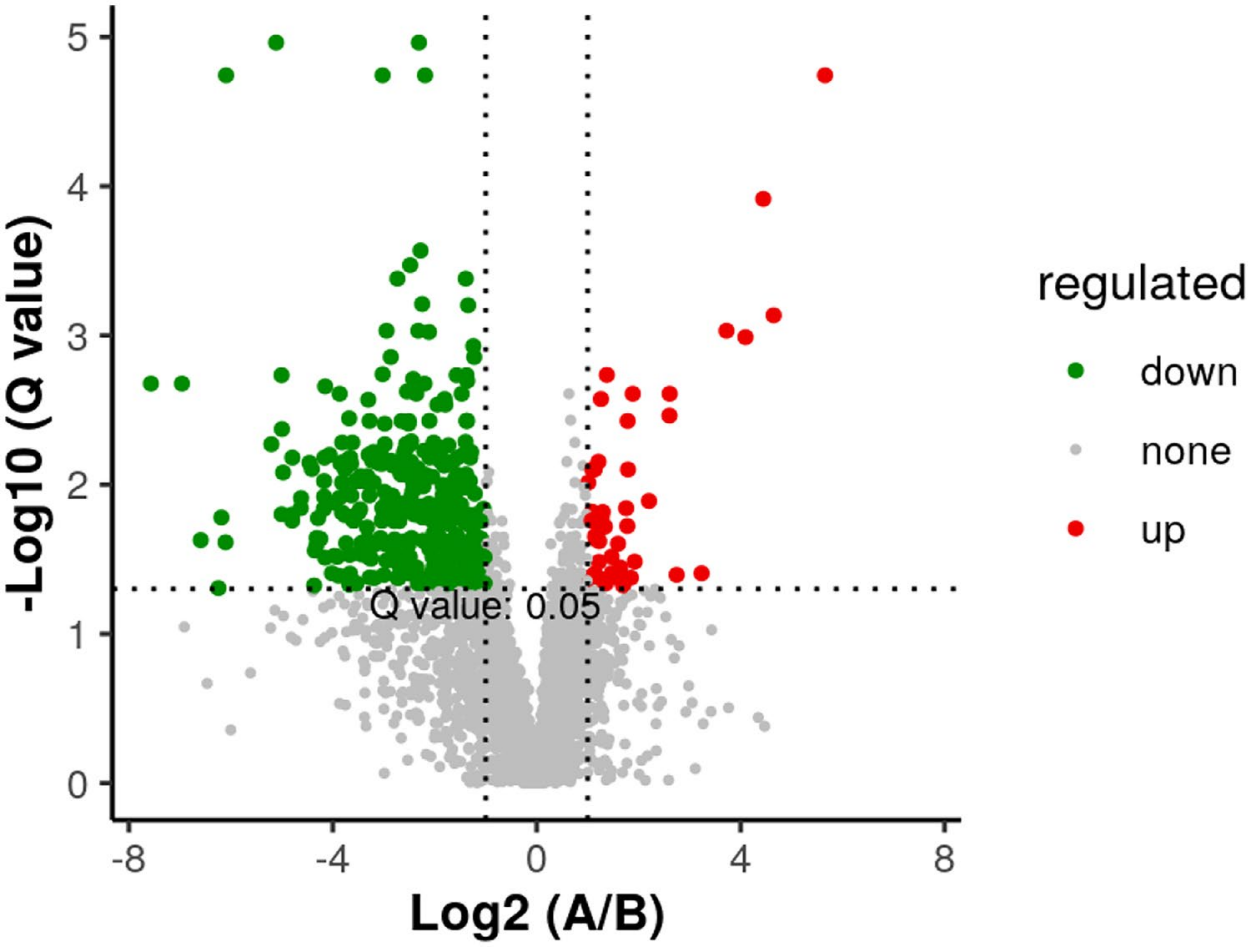
Volcano plot of differentially abundant metabolites. Note that the red dots represent up-regulation and the green dots represent down-regulation. **A** Triploid *Apostichopus japonicus* and **B** diploid *Apostichopus japonicus*

**Fig. 7:**
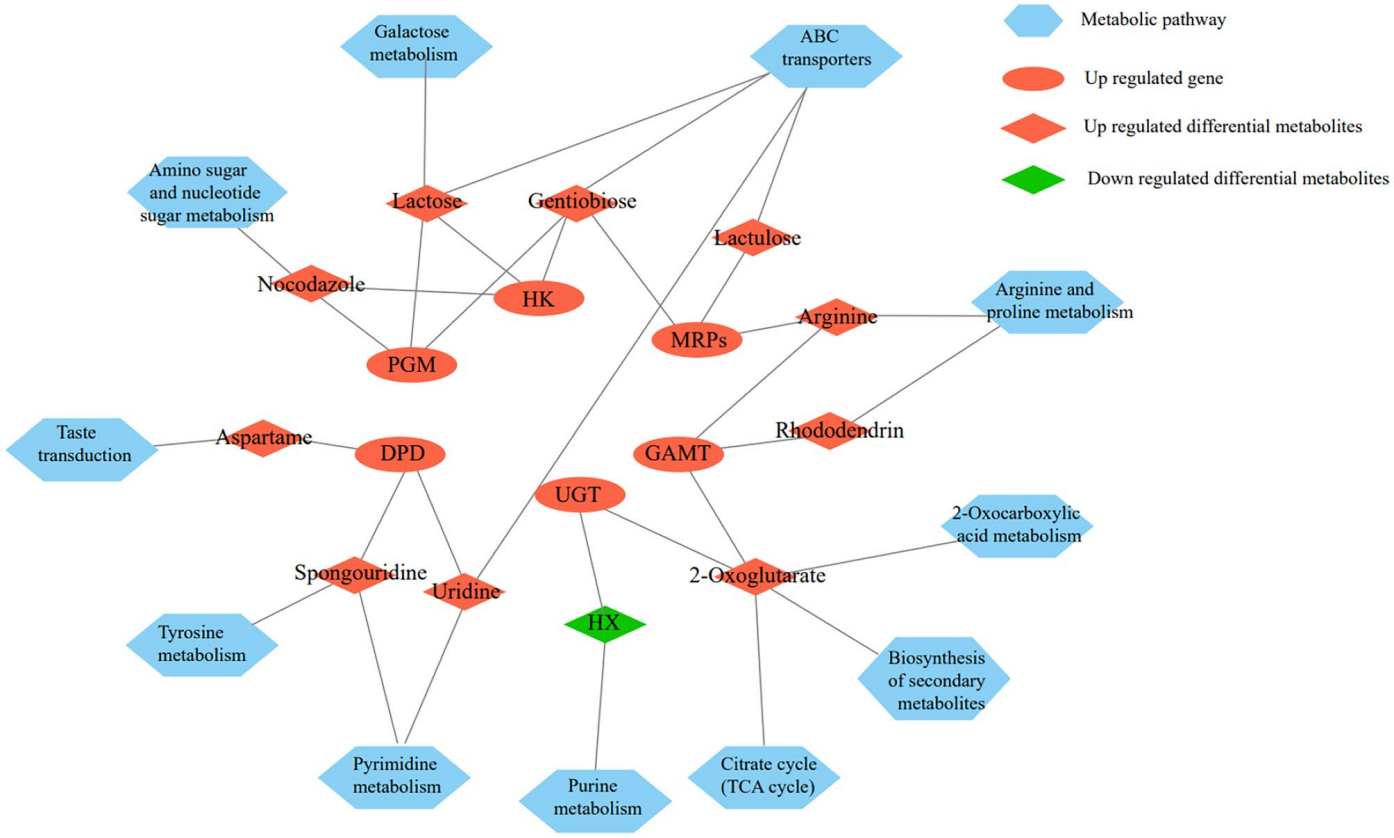
Results of combined transcriptomic and metabolomic analyses

We discovered 11 key significantly different metabolites (SDMs). Nocodazole, lactose, lactulose, gentiobiose, hypoxanthine (HX), 2-oxoglutarate, rhododendrin, arginine, uridine, spongouridine, and aspartame were among the SDMs (Figs. 6 and 7).

### KEGG Pathway Enrichment Results

According to the KEGG results, a total of 97 metabolic pathways were enriched. Significantly enriched in metabolic pathways (SDMs: 578, POS: 505, NEG: 73), porphyrin and chlorophyll metabolism (SDMs: 6, POS: 6, NEG: 0), fatty acid metabolism (SDMs: 9, POS: 8, NEG: 1), amino sugar and nucleotide sugar metabolism (SDMs: 14, POS: 14, NEG: 0), and fructose and mannose metabolism (SDMs: 6, POS: 4, NEG: 2) (Fig. S3).

### Combined RNA-Seq and Metabolome Analysis Results

We discovered that 6 DEGs may have regulatory relationships with 11 SDMs using a combined RNA-Seq and metabolome analysis, with 1 pair having negative regulation and 10 pairs having positive regulation (Fig. 7). We used GO enrichment analysis to look at the target genes of different metabolites. A total of 404 target genes (POS: 235, NEG: 165) were found to be enriched in 1151 items (POS: 666, NEG: 485). There were 572 items in terms of biological processes, mostly oxidation reduction processes (56 DEGs, POS: 28, NEG: 28) and metabolic processes (38 DEGs, POS: 20, NEG: 18), among others. There were 459 items in terms of molecular functions, mostly oxidative enzyme activity (40 DEGs, POS: 20, NEG: 20) and catalytic activity (44 DEGs, POS: 25, NEG: 19), among others. There were 120 cellular components, mostly cytosol (56 DEGs, POS: 30, NEG: 26) and cytoplasm (70 DEGs, POS: 50, NEG: 20), among others (Fig. S4); KEGG enrichment analysis of differential metabolite target genes revealed that 417 (POS: 246, NEG: 171) genes were enriched in 181 (POS: 92, NEG: 89) pathways, with purine metabolism (82 DEGs, POS: 41, NEG: 41) and pyrimidine metabolism (58 DEGs, POS: 33, NEG: 25) being the most enriched (Fig. S5).

## Discussion

The length of *A. japonicus* is an important economic characteristic. In this study, the length of diploid and triploid *A. japonicus* was significantly different under the same culture conditions. Among *A. japonicus* with a body length of 6–12 cm, the percentage of triploid *A. japonicus* was 4.46% higher than that of diploid *A. japonicus*. Among *A. japonicus* with a body length of 12–18 cm, the percentage of triploid *A. japonicus* was 1.37% higher than that of diploid *A. japonicus*. This was consistent with the triploid breeding results of other aquatic species (Peruzzi et al. 2018; Liu et al. 2018; Garnier-Géré et al. 2002). Following a comparative analysis, the aestivation time of triploid *A. japonicus* was shorter. This study proved that triploid *A. japonicus* had a better growth advantage.

Following sequencing analysis, we found that 6 DEGs had regulatory relationships with 11 SDMs, which jointly acted on 11 metabolic pathways (Fig. 7). The observed potential candidate genes mainly involved functions such as promoting growth and development, immune regulation, accumulation of carbohydrate, energy storage, and synthesis of beneficial metabolites; the differential metabolites were mainly concentrated in functions such as growth, immunity, carbohydrate synthesis, and taste improvement.

The results showed that triploid *A. japonicus* had certain growth advantages. We investigated whether the significant growth advantage of triploid *A. japonicus* was related to the enhancement of cell division, as the expressions of *CDK2*, *CDC45*, *ORC1*, and *histidine methyltransferase* were significantly higher in triploids. *CDK2* plays a key role in cell cycle regulation and participates in a series of biological processes. Existing research shows that *CDK2* may be involved in DNA damage and phosphorylation of protein interactions and antitumor activity (Tadesse et al. 2020; Spencer et al. 2013). *CDC45* can participate in the formation of the eukaryotic replication helicase and play an important role in the initial stage of DNA replication; thus, it can promote the cell cycle (Rios-Morales et al. 2019). Research has shown that *DNAJA1* after being activated by stable *CDC45* promotes the cell cycle (Yang et al. 2020). *ORC1* can participate in eukaryotic replication and maintain genome stability (De et al. 2019). The studies by Okano have proved that *ORC1* is essential for cell mitosis (Okano-Uchida et al. 2018). *Histidine methyltransferase* can regulate the differentiation of muscle cells and promote the growth and development of *A. japonicus* (Shu and Du 2021). The participation of genes in immune regulation contributes to the growth of triploid *A. japonicus*; the expressions of *UGT*, *GP96*, and *HSC70* are much higher in triploids. *UGT* is a glycoprotein attached to the endoplasmic reticulum cavity of microsomes. Bigo et al. (2013) indicated that *UGT* acted as a key metabolic protein in organisms to hinder the accumulation of toxic hydrophilic compounds. Primarily, it catalyzes the combination of endogenous or exogenous compounds with the cofactor uridine diphosphate glucuronic acid, thereby enhancing the polarity of lipophilic substrates and facilitating their excretion via urine or bile. For this reason, it has been proposed that triploid *A. japonicus* has a higher immunity and stronger stress resistance. The two heat shock proteins, *GP96* and *HSC70*, are generated at abnormal temperatures in an environment with extremely low oxygen content and oxidized free radicals during the bacterial infection process. They are able to repair denatured proteins, accelerate the recovery of normal proteins, and strongly protect cells when exposed to stress (Zininga et al. 2018). High temperature can induce the high expression of *HSC70*. The up-regulated expression of *HSC70* gene can protect against injury due to high temperature (Sun et al. 2016). *GP96* is highly expressed following increased temperature; therefore, it can resist high temperature (Tang et al. 2009). This may be the reason why the aestivation time of triploid *A. japonicus* is shorter. Also, they are important players in the processing of proteins in the endoplasmic reticulum (Cosin-Roger et al. 2018; Stricher et al. 2013). *HSC70* promote the processing and synthesis of proteins and benefits the growth of organisms (Liu et al. 2012); thus, it could be speculated that triploid *A. japonicus* has potential immune regulation ability than diploid *A. japonicus*. *DPD* is an enzyme mainly involved in the metabolism of pyrimidines in organisms. It contains the ability to metabolize the toxic pyrimidine analogue fluorouracil (5-FU). *DPD* is the rate limiting enzyme of fluoropyrimidine metabolism and can convert 5-FU and its metabolites into non-cytotoxic products and excrete them out of the body (Sharma et al. 2019). *DPD* expression was increased in triploid *A. japonicus*. Therefore, we speculate that triploid *A. japonicus* has a better ability to excrete toxins.

Carbohydrate accumulation can provide energy for triploid *A. japonicus*, and its related genes are *HK* and *PGM*, which are significantly up-regulated. *HK* is a pivotal rate-limiting enzyme in glycolysis; it is able to stimulate the utilization of glucose by cells and performs an extremely important function in energy metabolism (Patra et al. 2013). The energy provided by *HK* up-regulation may be used to resist the adverse effects at high temperature (Scaraffia and Gerez 2000). Variations in *HK* activity will give rise to changes in glycolysis and the formation of pentose phosphates, nicotinamide adenine dinucleotide phosphate hydrogen (NADPH), and glycogens (Anderson et al. 1971). *HK* with high activity is therefore beneficial to allow it to tackle hypoxia and high temperature stress. *PGM* participates in glycolysis and gluconeogenesis in vivo. Fragmentary or missing *PGM* will affect the synthesis of some elements of the cell wall (Morava 2014), and *PGM* deficiency greatly influences the glycosylation of proteins (Beamer 2015). Hence, high expression levels of related genes are speculated to be a vital influencing factor on the rapid growth and development of triploid *A. japonicus*. These results showed that the genes related to energy metabolism were up-regulated. *MRPs* and *GAMT* were significantly up-regulated in triploid *A. japonicus*. *MRPs* and *GAMT* are all related to the ATP energy supply. *MRPs* are members of the subfamily C of the ABC transporter superfamily and serve as efflux pumps for ATP (Zhang et al. 2015). They can pump antitumor drugs conjugated to glutathione, glucuronate, or sulfate out of cells. Arginine can generate creatine and glycocyamine under the action of *GAMT*. Studies have shown that creatine plays an important role in the conversion of ATP and participates in stabilizing a form of ATP (Joncquel-Chevalier et al. 2015). It is speculated that the up-regulation of *MRPs* and *GAMT* promotes the energy supply of triploid *A. japonicus*. Genes related to the synthesis of beneficial metabolites were up-regulated. *CBRs* is a type of oxidoreductase protein, which is widely found in bacteria, fungi, yeasts, animals, and plants. With coenzyme NAD(P) + and NAD(P)H as the electron acceptor and donor, respectively, *CBRs* can specifically catalyze the interconversion between ketone (aldehyde) and alcohol, and contribute to the synthesis of valuable hydroxy compounds and metabolites (Forrest and Gonzalez 2000).

According to the combined analysis of transcriptome and metabolome, we screened 11 key SDMs which are related to growth and development (SDMs: nocodazole, rhododendrin, 2-oxoglutarate, arginine, lactulose, and HX), immunity (SDMs: uridine and spongouridine), and taste (SDMs: lactose, gentiobiose, and aspartame), respectively. Nocodazole, rhododendrin, 2-oxoglutarate (AKG), arginine and lactulose are positively regulated by the DEGs *HK* (SDM: nocodazole), *PGM* (SDM: nocodazole), *GAMT* (SDMs: rhododendrin and arginine), and *MRPs* (SDMs: arginine and lactulose). HX is negatively regulated by *UGT*. They are involved in the metabolic pathways of amino sugar and nucleotide sugar metabolism, arginine and proline metabolism, TCA cycle, 2-oxocarboxylic acid metabolism, biosynthesis of secondary metabolites, ABC transporters, and purine metabolism. Nocodazole is a mitotic blocker in the same class as colchicine, and it can induce the arrest of cell division in the *M* phase and lead to cell synchronization (Cooper et al. 2006). Attia found that nocodazole was a germ cell mutagen, which induced mutation lethality in male germ cells (Attia et al. 2015). Based on its ability to kill germ cells, it was inferred that the up-regulated expression of such metabolites is related to the sterility of triploid *A. japonicus*. According to research carried out by Kim (Kim et al. 2019), rhododendrin significantly enhanced the activity of dopaminergic neurons. The metabolite AKG can promote growth. Studies have shown that AKG can promote the synthesis of muscle protein (Pierzynowski and Sjodin 1998). In piglets with slow perinatal growth, AKG as an energy donor can promote bone metabolism (Tomaszewska et al. 2021). Arginine is a basic component of various proteins, and its precursor can regulate cell proliferation, differentiation, and homeostasis (Bulau et al. 2006). Research carried out by Deng indicated that lactulose can promote enterocinesia (Deng et al. 2021). It may accelerate the intestinal absorption of nutrients and provide more energy for the body. HX is an important purine alkaloid that degrades fats. The down-regulation of HX benefits the development of triploid *A. japonicus*. Therefore, these metabolites may lead to the rapid growth and development of triploid *A. japonicus*. Uridine and spongouridine are positively regulated by *DPD*, and they are jointly involved in the metabolic pathways of ABC transporters, pyrimidine metabolism, and tyrosine metabolism. Uridine is a ribose extracted from nucleic acids. Walker revealed that uridine reduced cell apoptosis, and prevented mitochondrial DNA deletion and mitochondrial depolarization (Walker and Venhoff 2005). It has been found that spongouridine, a metabolite with antibacterial ability, inhibited bacteria and fungi to some extent (Hamoda et al. 2021). It is speculated that the up-regulation of these metabolites can enhance the antibacterial immunity of triploid *A. japonicus*. Lactose, gentiobiose, and aspartame are positively regulated by the DEGs *HK* (SDMs: lactose and gentiobiose), *PGM* (SDMs: lactose and gentiobiose), and *DPD* (SDM: aspartame). They are involved in the metabolic pathways of galactose metabolism, ABC transporters, and taste transduction. Lactose, one of the sources of dietary energy, can effectively improve the quality of products, maintain the color of products, and increase the overall sugar content without making the products too sweet and greasy. Luo and Jiang (2006) proved that lactose improved the flavor of shredded squid and reduced water activity. As a type of functional oligosaccharide, gentiobiose can effectively improve food flavors, and function as an immune regulator in vitro (İspirli et al. 2019). Aspartame is an artificial dipeptide sweetener that is able to affect a variety of cells and tissues (Choudhary and Pretorius 2017). It is presumed that the up-regulated expression of these metabolites can enhance the nutritional value of triploid *A. japonicus*, and optimize flavor and taste.

Compared with diploid *A. japonicus*, triploid *A. japonicus* had more advantages in terms of gene regulation ability, error-tolerant rate of gene expression, immunity, growth and development, stress resistance, energy conversion rate, adaptability to a harsh living environment, edible value, and taste. Our results further enrich the biological data of triploid *A. japonicus* and provide useful resources for genetic improvement of this species.

## Supplementary Information

Below is the link to the electronic supplementary material.

Supplementary file1 (DOCX 711 KB)
