# Supplementary material for: Transcriptomic and Metabolomic Analyses Provide Insights into the Growth and Development Advantages of Triploid Apostichopus japonicus
Source: Mar Biotechnol (NY). 2022 Feb 5;24(1):151–62. doi: 10.1007/s10126-022-10093-4 (PMC8940865; doi:10.1007/s10126-022-10093-4)
Supplement: Supplementary file 1 — Supplementary file1 (DOCX 711 KB) [file 10126_2022_10093_MOESM1_ESM.docx]

**Supplementary materials**

**Figure S1.** Differentially expressed genes GO enrichment analysis top50


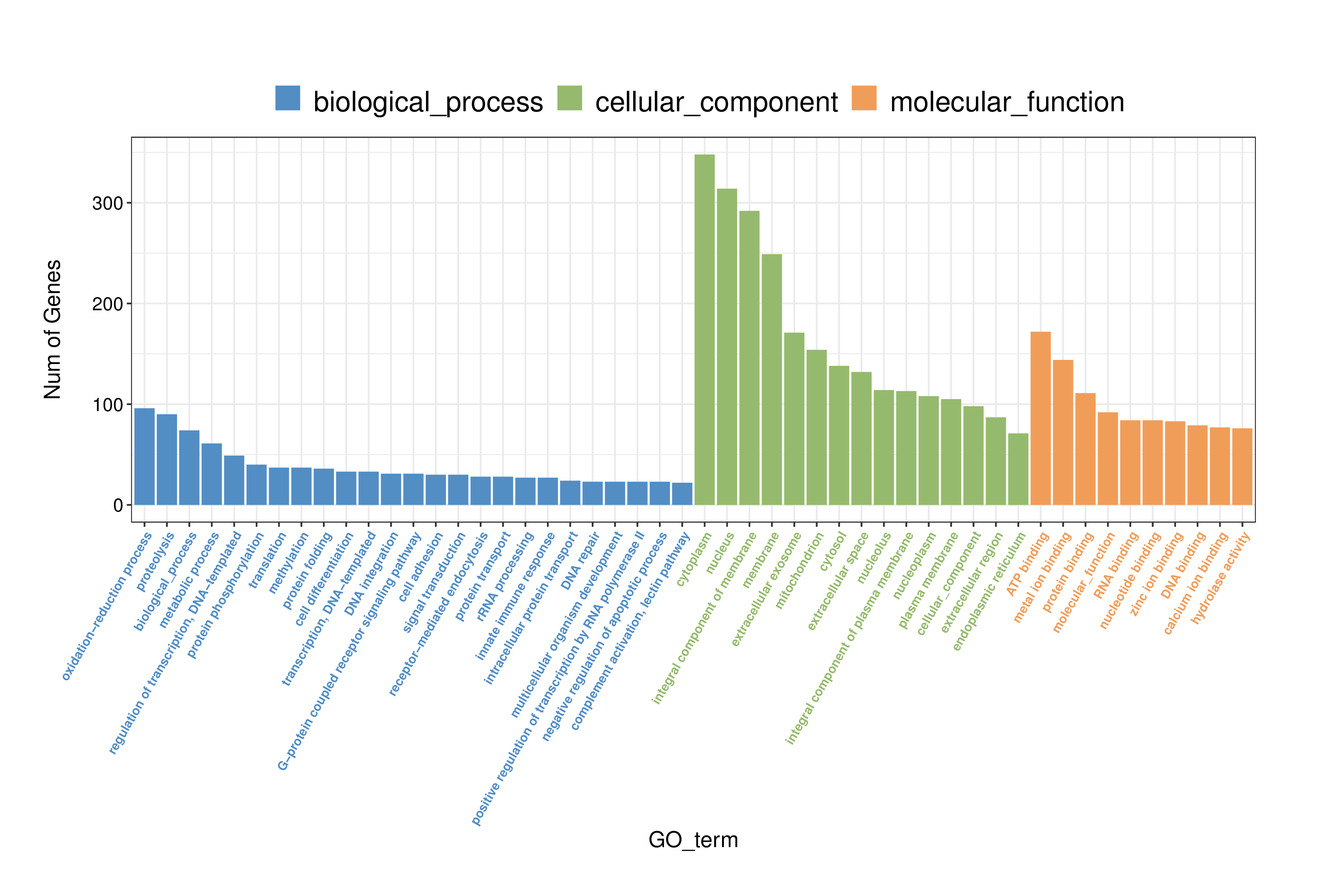


**Figure S2.** Differentially expressed gene KEGG enrichment analysis top20

Note: The X axis in the figure is the enrichment score. The larger the bubble, the more differential protein coding genes contained in the pathway. The color of the bubble changes from blue-purple-green-yellow-red, which means that the smaller the p-value is, The greater the degree of significance.


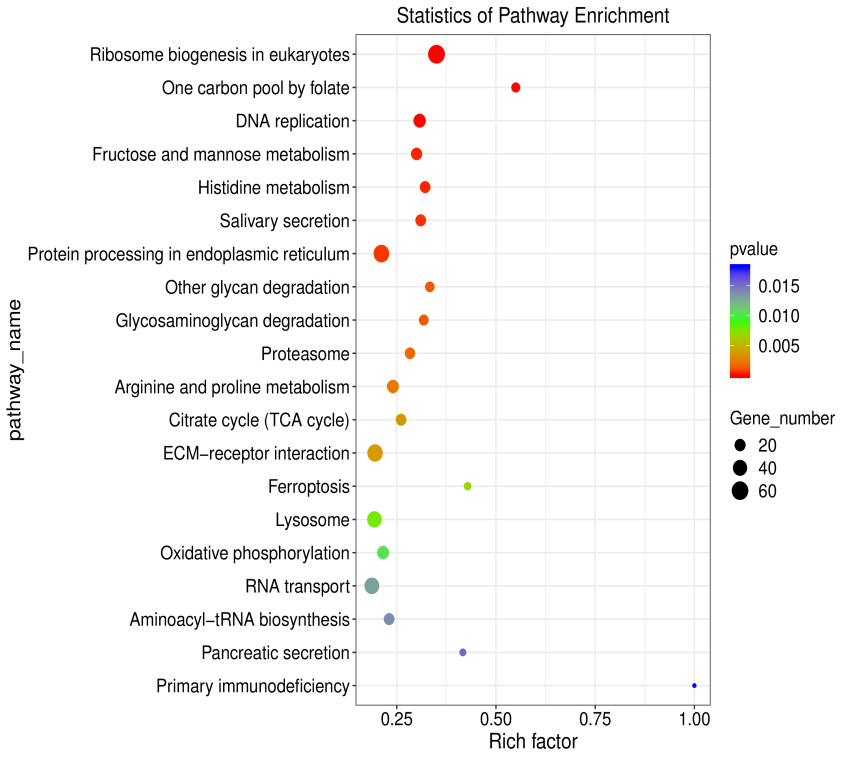


**Figure S3.** Classification diagram of KEGG pathway

Note: The second-level item of Kegg Pathway was taken as the abscissor, and the metabolite quantity of the corresponding item was taken as the ordinate. The Kegg Pathway level 1 entries were displayed and distinguished by different colors.


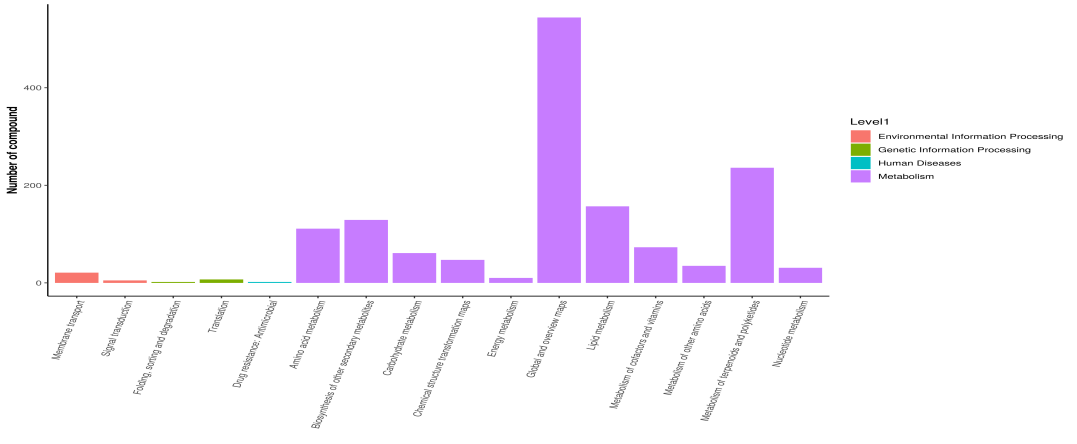


**Figure S4.** Classification diagram of GO pathway by combined analysis of transcriptome and metabolome


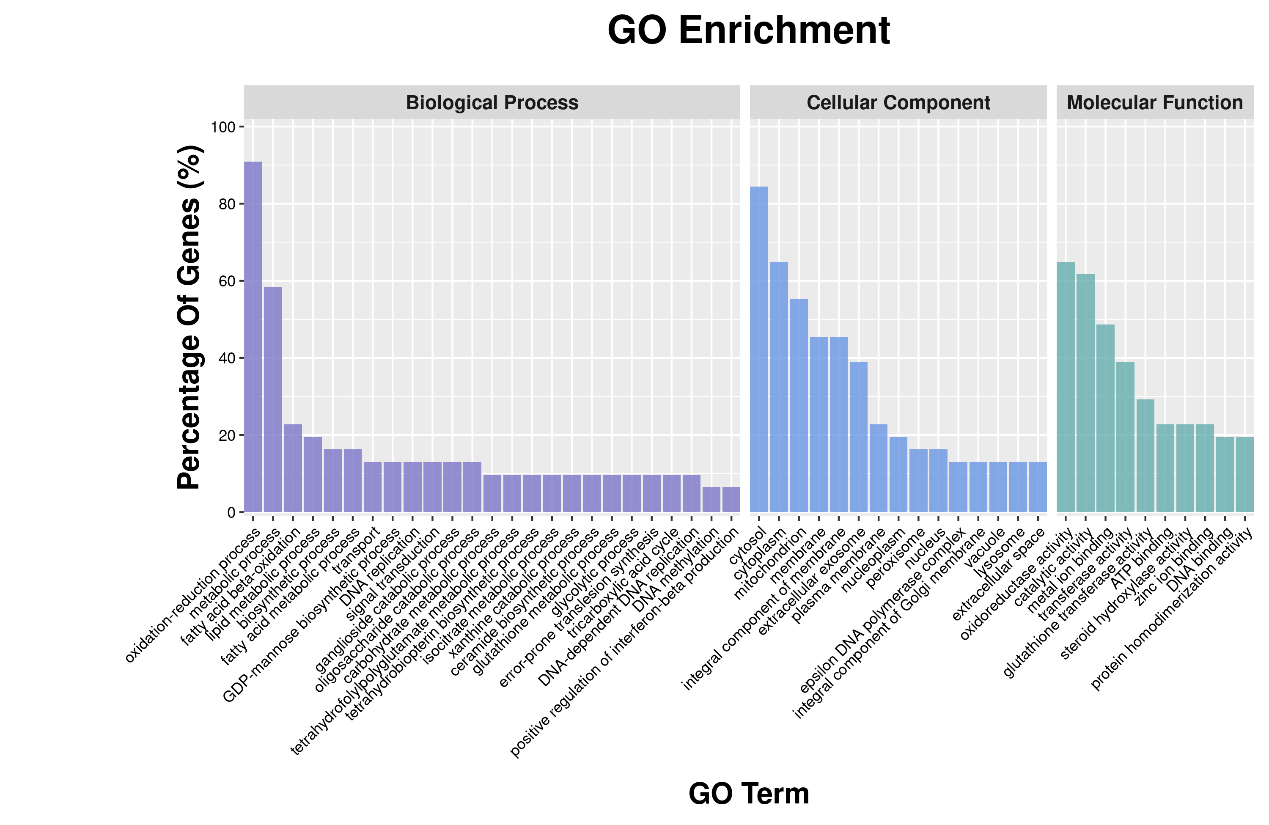


**Figure S5.** Classification diagram of KEGG pathway by combined analysis of transcriptome and metabolome


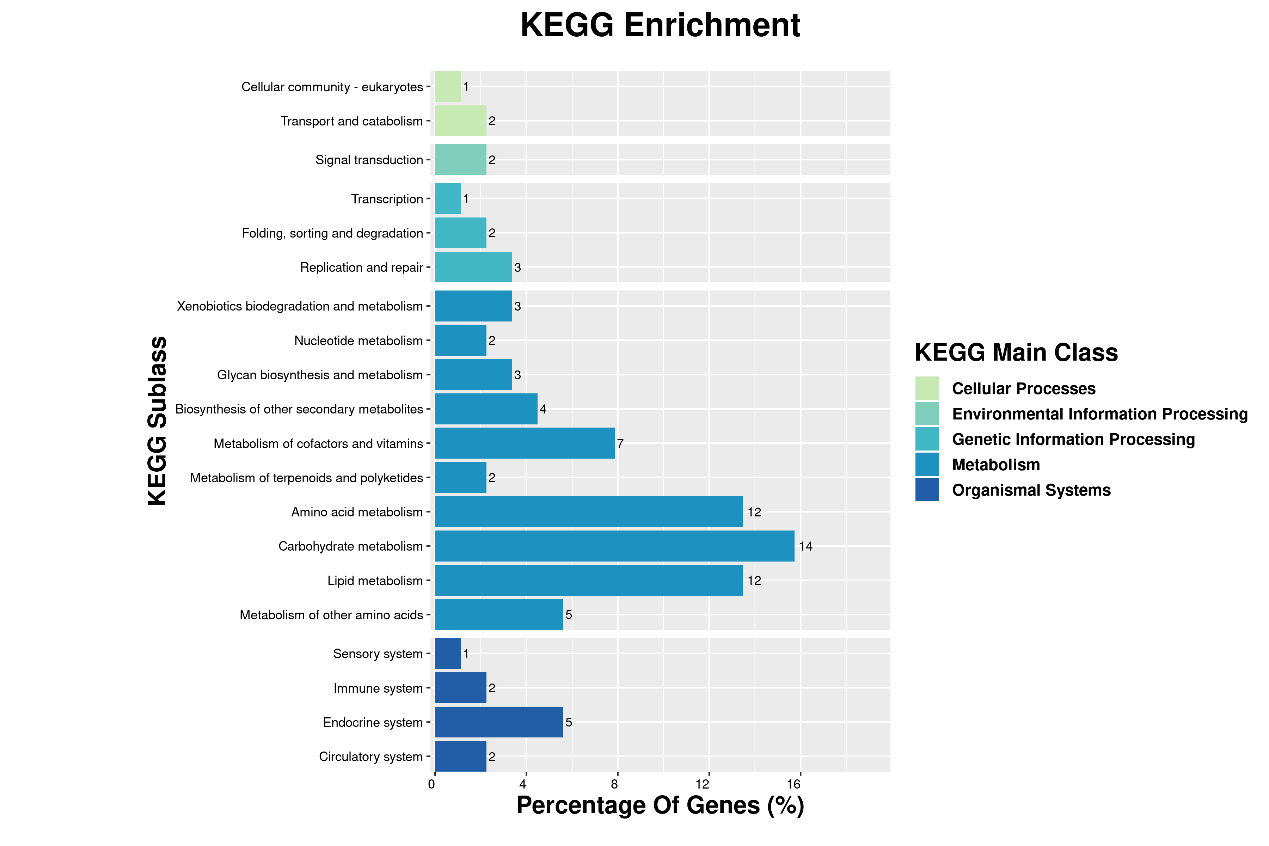


**Figure S6.** Ploidy detection diagram of diploid and triploid *Apostichopus japonicus* (*Apostichopus japonicus* cultivated in 2019)

Note: A. Diploid *Apostichopus japonicus* ploidy test results; B. Triploid *Apostichopus japonicus* ploidy test results

**
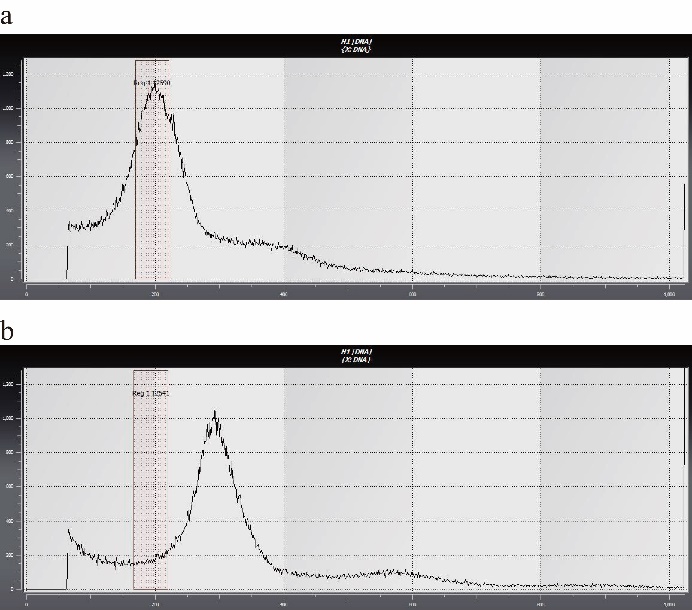
**

**Table S1.** Triploid and diploid *Apostichopus japonicus* growth data

| Group | Measurement indicators | 12~18cm | 6~12cm | 0~6cm |
| --- | --- | --- | --- | --- |
| Diploid group | The number of samples | 18 | 27 | 2127 |
|  | Average body length(cm) | 13.51±1.32 | 7.57±1.99 | 4.01±1.23 |
|  | Average weight(g) | 76.77±4.27 | 33.34±3.24 | 2.58±0.98 |
|  | Quantity percentage (%) | 0.83 | 1.24 | 97.93 |
| Triploid group | The number of samples | 11 | 29 | 470 |
|  | Average body length(cm) | 13.42±1.43 | 10.55±2.07 | 2.43±0.89 |
|  | Average weight(g) | 71.27±3.46 | 47.66±2.77 | 1.96±0.71 |
|  | Quantity percentage (%) | 2.16 | 5.69 | 92.15 |

**Table S2.** Data quality processing results of transcriptome sequencing data of triploid *Apostichopus japonicus* and control group *Apostichopus japonicus*

| Sample | raw  reads | Raw  bases | Clean  reads | Clean  bases | Valid  bases | Q30 | GC |
| --- | --- | --- | --- | --- | --- | --- | --- |
| Diploid 2n-1 | 36.23M | 5.44G | 32.85M | 4.93G | 90.66% | 98.01% | 41.00% |
| Diploid 2n-2 | 44.11M | 6.62G | 40.39M | 6.06G | 91.56% | 98.14% | 42.00% |
| Diploid 2n-3 | 37.16M | 5.57G | 33.55M | 5.03G | 90.28% | 98.11% | 43.00% |
| Triploid 3n-1 | 44.14M | 6.62G | 41.10M | 6.17G | 93.12% | 98.18% | 42.00% |
| Triploid 3n-2 | 51.01M | 7.65G | 46.34M | 6.95G | 90.85% | 98.16% | 42.00% |
| Triploid 3n-3 | 46.68M | 7.00G | 37.80M | 5.67G | 80.98% | 98.40% | 42.00% |
